# Current status and future trends for pork production in the United States of America and Canada

**DOI:** 10.5713/ab.24.0055

**Published:** 2024-04-01

**Authors:** M. Todd See

**Affiliations:** 1 Department of Animal Science, North Carolina State University, Raleigh, NC 27695-7621, USA

**Keywords:** Pigs, Pork, Pork Production, Production System, Swine

## Abstract

Pork production is a significant agricultural enterprise in the United States and Canada. The United States is the third-largest global producer of pork and Canada ranks seventh in pork production. The North American Free Trade Agreement and its successor, the U.S.-Mexico-Canada Agreement, have facilitated trade and integration between the two countries. The majority of production systems are modern and intensive, characterized by large vertically integrated farms using advanced technologies. Both nations benefit from their status as major producers of feed grains, with the United States leading in corn and soybeans, while Canada excels in canola and barley production. The regulatory frameworks for food safety, animal welfare, and environmental stewardship differ slightly, with the FDA and USDA overseeing these aspects in the United States, and Health Canada and the Canada Food Inspection Agency in Canada. The United States and Canada also have well-established distribution networks for pork products, relying on both domestic and international markets. Export markets play a crucial role, with the United States being a major importer of Canadian pigs, and both countries exploring opportunities in Asia. Despite a rise in global demand, domestic pork consumption trends differ, with per capita consumption remaining stable in the USA and declining in Canada. Changing consumer preferences, including a demand for ethically raised and locally sourced pork, may influence production practices. Future trends in pig production include a focus on consumer concerns, sustainability, disease prevention, reduction of antimicrobial use, and advancements in technology. The industry is adapting to challenges such as disease outbreaks and changing regulations, with a strong emphasis on animal welfare. Labor and workforce considerations, along with advancements in technology and automation, are expected to shape the efficiency of pork production in the future.

## PIG PRODUCTION IN THE UNITED STATES AND CANADA

**Figure 1:**
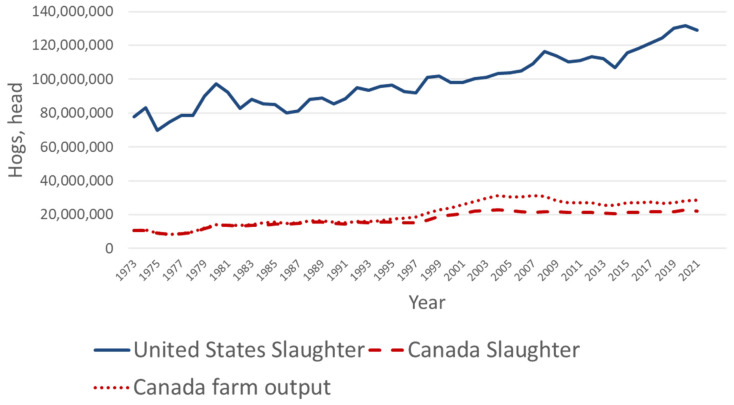
Trends of hog production, head slaughtered, in the United States and Canada [2,3].

Pork production is an important agricultural sector in both the United States and Canada. The United States has a larger pork industry in terms of production scale compared to Canada, reflecting the larger population and market size of the United States of America (USA). The United States is the third largest producer of pork following China and the European Union and Canada is the seventh largest producer after Brazil, Russia and Vietnam [1]. Production has been steadily increasing over time in both countries (Figure 1) [2,3] due to growing global demand for pork products. Hogs produced in the USA and Canada has increased forty percent (Figure 1) [2,3] over the last fifty years with over 150 million head harvested in 2021. Growth in pork production has resulted in both the United States and Canada becoming leading exporters of pork [2,3].

**Figure 2:**
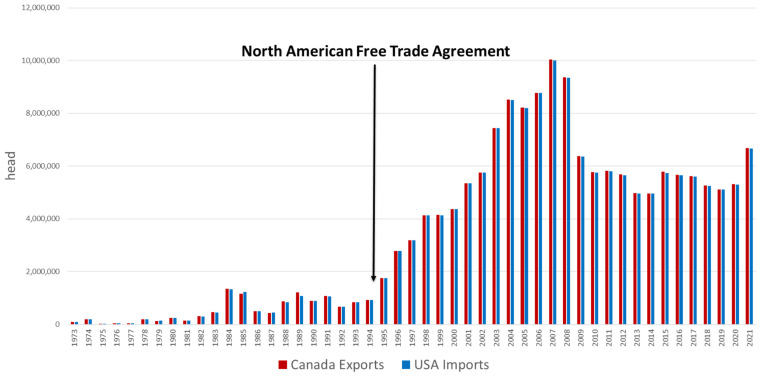
Canadian live hog exports and United States (USA) live hog imports [6].

**Figure 3:**
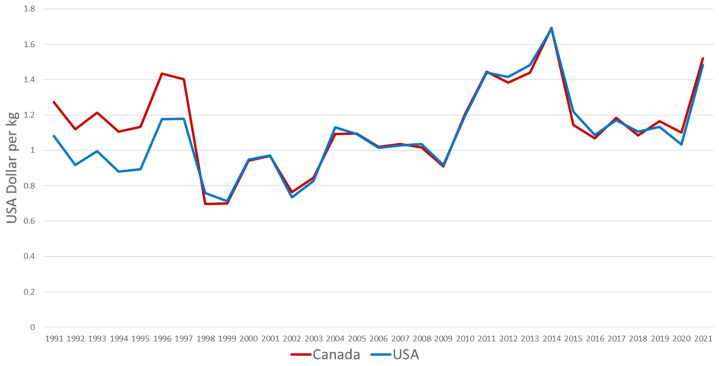
Pork prices in the USA and Canada [6].

Pork production systems in the USA and Canada are closely linked due to trade and market integration [4]. Import duties were dropped and non-scientific barriers to trade were significantly reduced after the passage of the North American Free Trade Agreement (NAFTA) in 1994 which continued until July 1, 2020 when replaced by the U.S.-Mexico-Canada Agreement (USMCA) [5]. The USMCA preserved zero-tariff trade and further strengthened agricultural trade between the USA, Canada and Mexico [5]. The impact of NAFTA and USMCA is clearly depicted in Figure 2 where exports of live hogs from Canada are nearly equal to the imports of live hogs into the USA [6]. Due to these trade agreements, pork prices are also tightly aligned between the USA and Canada (Figure 3) [6]. Nearly one-fourth of the pigs born on Canadian farms are sent to the USA for slaughter (Figure 2) [6]. The USA is the largest importer of Canadian pigs and pork while Mexico is the largest importer of USA pork. The import of Canadian Pigs into the USA accounts for over 75% of the world trade of live hogs [6].

**Figure 4:**
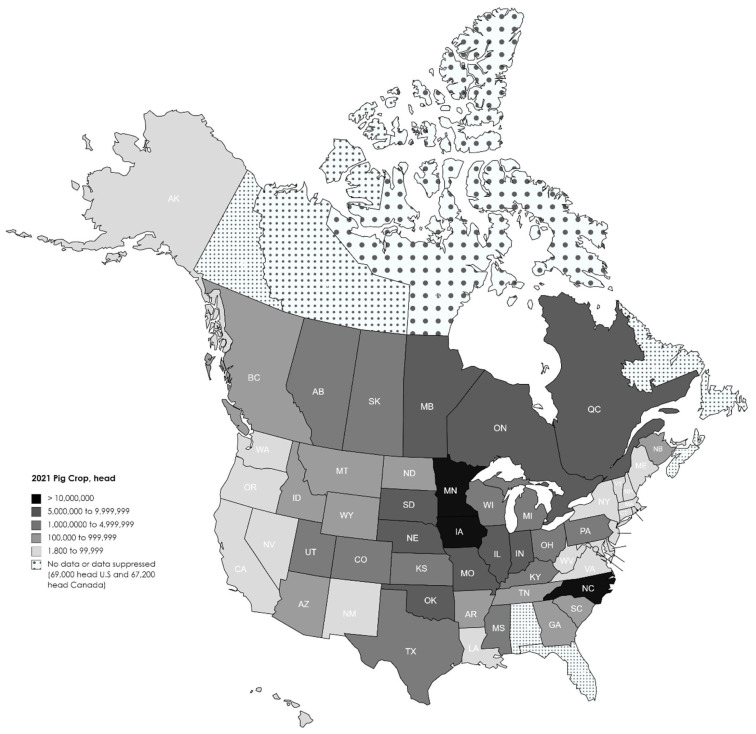
Location of pig production in the United States and Canada [2,3].

**Figure 5:**
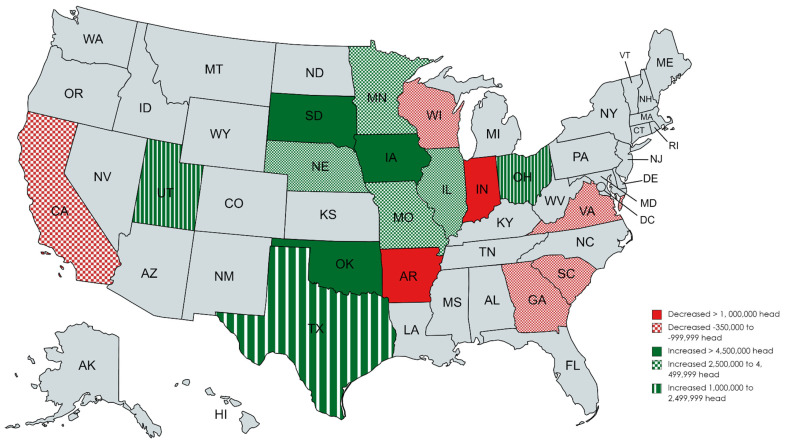
Changes in location of United States pig production between 1997 and 2022 [2].

Pork production is widely distributed across the fifty states and ten Canadian provinces (Figure 4) [2,3]. However, pork production is also more concentrated in the central part of the North American continent (Figure 4) [2,3] due to the cereal grain and grain byproduct produced in this region. More than seventy percent of the pig crop was produced in just ten states and provinces in 2021 (Table 1) [2,3]. The location of pig farms in the United State continues to evolve (Figure 5) [2]. In general growth of pig production has been reported in the central USA with declines on the coast (California, Virginia, South Carolina, and Georgia). Over the last twenty-five years the largest increases in pig production were observed in Iowa, South Dakota and Oklahoma while declines were reported in Indiana and Arkansas. This is in contrast to the growth in production that occurred from 1980 to 2000 which took place in North Carolina, the southwest and western parts of the USA [7].

Significant investments in research and development have led to advanced technologies and practices in pig farming. This includes improved genetics, nutrition, housing systems, and disease management techniques which have helped to increase productivity and efficiency [8]. Production is also dominated by large vertically integrated and/or vertically coordinated production systems (Table 2) [9]. These producers commonly utilize two and three site production systems. The United States has a more extensive range of farm types and sizes than Canada. In 2021, there was a reported 67,000 farms averaging 1,080 hogs per farm in the USA and 7,635 farms averaging 1,820 hogs per farm in Canada [2,10]. Collectively these data suggest greater variability in USA farm sizes with a significant number of smaller farms producing a small proportion of total pigs.

While both countries utilize a mix of production systems, the USA and Canada predominantly have modern, intensive production systems where large numbers of pigs are housed in environmentally controlled facilities, allowing for efficient and controlled production. These systems also operate within an integrated supply chain model. This involves collaboration among pig farmers, processors, packers, distributors, and retailers to ensure efficient production, processing, and distribution of pork products. Highly developed and large-scale pig farms allow for economies of scale, where larger operations can benefit from lower production costs per unit. Large pig farms have efficient production systems, advanced infrastructure, and better access to resources like feed, veterinary services, and equipment. Pig farming also benefits from the presence of agricultural universities, extension services, and industry associations which further support knowledge sharing and trained professionals, including veterinarians, nutritionists, and researchers.

## FEED AVAILABILITY

The United States and Canada are major producers of feed grains and oilseeds which provides a cost advantage to pig producers. Additionally, the availability of high-quality, locally sourced feed contributes to the overall health and productivity of the animals. While both countries produce large quantities of feed grains, there are some differences in terms of production and specific crop preferences. Factors such as crop production, climate, and feed ingredient availability can influence feed formulation practices and strategies employed by pork producers in each country.

The United States is the world's largest producer of corn and has a highly developed corn industry. United States corn accounts for nearly one-third of world corn production and 95% of total USA feed grain production [11]. Corn is a staple feed grain for livestock in the USA, with about forty percent of the crop being used for animal feed [12]. Corn production in Canada is relatively smaller compared to the USA. Canadian corn production is concentrated in the southern regions of Ontario and Quebec [3]. In 2022, the USA produced 354 million metric ton (MMT) of corn compared to 14.5 MMT in Canada [13].

The USA is the world’s second largest producer and exporter of soybeans [13]. Soybeans account for about 90 percent of USA oilseed production with nearly half of the soybean production exported annually [14]. In 2022, the USA produced 116 MMT of soybeans compared to 6.7 MMT in Canada [13].

Canada is one of the largest producers of canola and barley in the world [13] producing 18.2 MMT of canola and 10 MMT of barley in 2022. Canola and barley production is concentrated in the western provinces, particularly Alberta and Saskatchewan. Canadian barley is used for various purposes, including feed grain for livestock and malt production. Canola and barley production in the USA is relatively smaller with 2022 production of 1.79 MMT and 3.8 MMT of canola and barley, respectively [13]. Canola and barley is mainly grown in the northern regions of the USA, such as North Dakota and Montana.

Wheat ranks first amongst crops in Canada (33.8 MMT) and third in the United States (49 MMT) [13], but a substantial portion of the wheat produced is of the high-quality milling type used for human consumption. However, some lower-grade wheat is also used for feed purposes.

## REGULATORY FRAMEWORK

The regulatory frameworks governing pork production have similarities but also differ between the United States and Canada. Each country has its own set of regulations and standards related to food safety, animal welfare, environmental stewardship, and labeling requirements. Both the USA and Canada benefit from rigorous food safety regulations and quality control measures which ensure the production of safe and high-quality pork products and provide an advantage in international trade. These include government oversight, industry-led programs, and third-party certifications.

In the United States the Food and Drug Administration (FDA) oversight is very broad and is responsible for the oversight, safety and efficacy of veterinary drugs and livestock feed [15]. However, even though the FDA is responsible for food safety as implied in the name that does not include meat, poultry and egg products [15]. The safety of pork along with other meats and eggs is the responsibility of the United States Department of Agriculture Food Safety and Inspection Service [15] and oversight of veterinary biologics is the responsibility of the USDA Animal and Plant Health Inspection Service [15]. The USDA is led by the Secretary of Agriculture and the FDA by the Secretary of Health and Human Services.

In Canada these roles are also performed by two separate agencies which are both part of Health Portfolio [16] which is led by the Minister of Health. Health Canada evaluates and monitors the safety, quality, and effectiveness, sets standards, and promotes the prudent use of veterinary drugs administered to food-producing and companion animals [16]. The Canada Food Inspection Agency is dedicated to safeguarding food from both animals and plants [17].

## PORK EXPORT

The United States and Canada have a well-established distribution network and infrastructure for pork products, both domestically and internationally. Both countries have access to diverse markets, including a large domestic consumer base and export opportunities. This enables pig farmers to efficiently reach consumers and maximize their market potential.

Both the USA and Canada rely on export markets for their pork products and these markets have been expanding to meet the growing demand worldwide. However, profitable pork production in Canada is more reliant on export markets than in the USA. In 2022, exports of pork and pork products accounted for 27.5% and 63.2% of total production from the USA and Canada, respectively [1,10]. In 2022, the value of pork exports from the USA was $7.7 B USD and from Canada $3.5 B USD [1,10]. Significant exports occur between the USA, Canada and Mexico (Table 3) [1,10] due to the USMCA [5]. Significant trading partners for pork outside of North America include Japan, China, South Korea and the Philippines (Table 3) [1,10].

Exports of pork from the USA and Canada are influenced by factors such as international trade policies, global demand, and disease outbreaks in other countries. Access to international markets and trade agreements will play a crucial role in shaping future export opportunities. Future trends will depend on factors such as global demand, trade policies, and competition from other pork-producing countries like China, Brazil, and European countries.

The pork industries of the United States and Canada will continue to explore and expand export opportunities in emerging markets, particularly in Asia, as rising incomes and changing dietary preferences increase demand for pork. Because of steep competition among the pork exporting countries, specific consumer preferences will should be considered carefully, to increase the amount of pork exported to specific countries [18]. In addition, Canadian pork industry is expected to focus on diversifying its export markets to reduce reliance on the USA.

## DOMESTIC PORK CONSUMPTION

**Figure 6:**
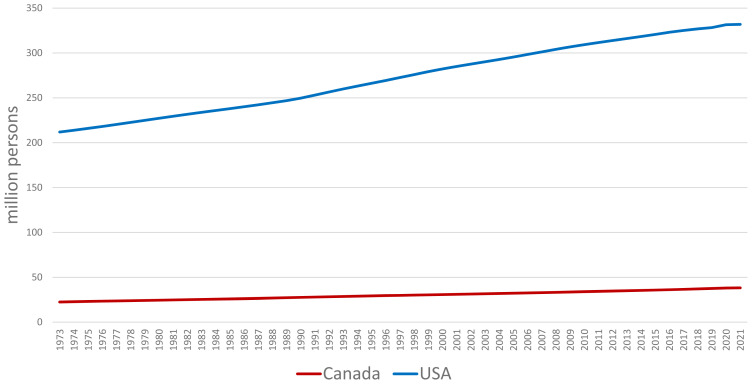
Growth in domestic demand for pork in the United States is due to increasing population [19].

**Figure 7:**
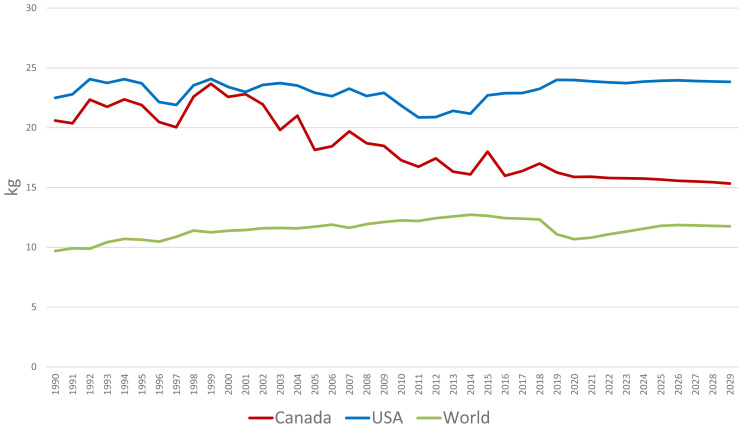
Per capita consumption of pork is steady in the United States but declining in Canada [19].

The demand for pork is expected to continue to rise in the USA and Canada due to population growth (Figure 6) [19]. Per capita consumption of pork in the USA has remained relatively stable, reflecting pork's popularity as a protein source (Figure 7) [19]. However, per capita consumption of pork in Canada has been declining over the last twenty years (Figure 7) [19].

While the vast majority of USA and Canadian pork is produced in commercial systems and consumed domestically consumer preferences are showing shifts towards ethically raised, antibiotic-free, and locally sourced pork, which could influence production practices. There are also domestic opportunities for pork products that meet specific quality standards and have value added to be sold in higher margin markets. Producers may need to adapt to changing consumer demands to remain competitive in the market with other protein sources.

## FUTURE TRENDS

Farmers in the USA and Canada initially evolved their outlook from being producers of hogs to producers of pork and protein with a focus on quality and food safety [20]. This attention to the consumer has continued and accelerated. Pork producers are increasingly concerned with and responding to consumer demands and concerns. A Pork Checkoff-funded study [21] found that nearly one-third of consumers said they have reduced or plan to reduce pork consumption due to concerns about nutrition, safety and ethics of raising pork. In response the National Pork Board in the USA has leveraged their Real Pork [21] program to increase consumer confidence in pork. The National Pork Board has also partnered with a consortium of universities (Iowa State University, North Carolina Agricultural and Technical University, North Carolina State University, University of Georgia and University of Minnesota) to create the Real Pork Trust Consortium to i) research pressing topics to answer consumer questions, ii) communicate pork research finding in a relatable and relevant way, and iii) train individuals to share evidence-based information about the pork industry [22]. Focus areas today and in the future include sustainability, animal well-being, food safety, public health, disease, labor and workforce. Process improvements in these areas may increase cost of production which will result in continued focus on efficiency of production and the adoption of new technologies.

Like the global pork industry disease outbreaks (e.g. PRRS and PEDv) continue to challenge producers and have a significant impact on pork production. The industry will continue to focus on disease prevention, biosecurity measures, and research to address emerging threats like African swine fever or other diseases that may affect production. Proactive measures to safeguard herd health will be crucial for future production. African swine fever has not been detected in Canada or the USA [23]. Classical swine fever and Foot and Mouth Disease were eradicated in the U.S. in 1978 and 1929, respectively [24]. The United States and Canada have agreed to continuation of safe trade in the event of African Swine Fever in either country [25] Due to the global transmission of African Swine Fever there is a current focus on promotion of awareness, border protection, biosecurity, surveillance systems, emergency plans and vaccine candidates to protect the USA and Canada pork industry. The development of advanced systems for biosecurity plans and visualization of biosecurity infrastructure is having a positive impact on swine health [26].

**Figure 8:**
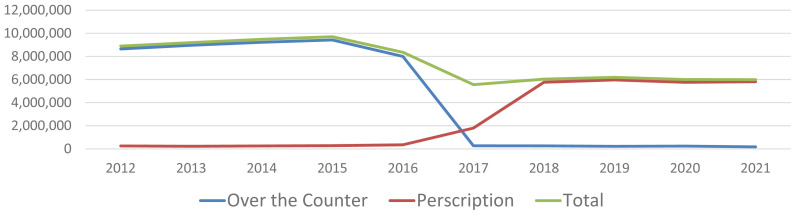
Medically important antimicrobial drug use (kg) in USA food producing animals by dispensing status [27].

In contrast to improving animal health is the global trend for the reduction of antimicrobial use. Methods that establish and maintain herd health allow for the reduction of antimicrobials in food production but not the complete elimination. There has been a major focus on the judicious use of antibiotics resulting in a reduction of antibiotics provided to food producing animals (Figure 8) [27]. The observed reduction occurred due to the implementation by the FDA of Guidance for Industry #213 [28] which transitional medicinally important antimicrobials that are used in feed or drinking water to veterinary oversight and eliminated the use of these products in animals for production purposes. In 2023, the FDA implemented Guidance for Industry #263 [29] which removed all over-the-counter antimicrobials. Many researchers are evaluating and seeking alternatives to antimicrobials.

Growing consumer demand for sustainable and ethically produced pork is driving efforts to improve environmental practices, animal wellbeing standards, and the reduction of the carbon footprint. A retrospective report found that over the last fifty years producers in the USA are producing pork on a per unit basis that uses 75.9% less land, 25.1% less water and has 7.7% less potential for global warming [30]. While these are tremendous improvements, research and adoption of technologies that enhance the soil, water, and air continues.

**Figure 9:**
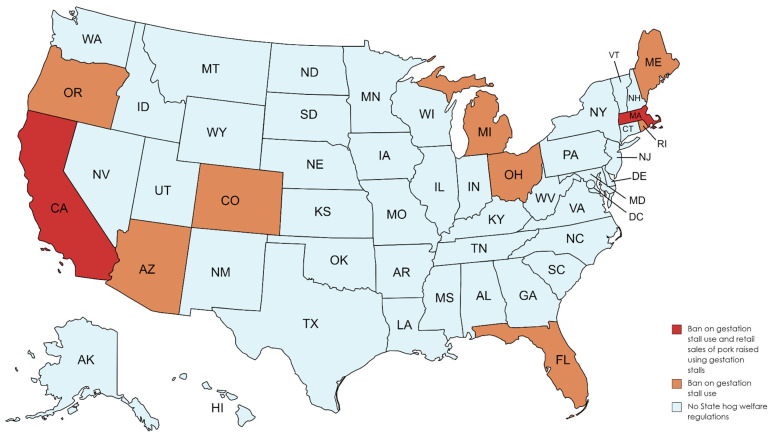
States with laws on how sows are housed [31].

Pork producers are also facing increased scrutiny and regulations related to confinement systems that may influence production practices and require investments in infrastructure. Changes in regulations and policies related to animal care standards are expected to influence the future of pork production. In the USA, nine states have passed regulations banning the use of gestation stalls on farms in those states (Figure 9) [31]. The California and Massachusetts laws also ban the sale of pork products raised using gestation stalls in other states [31]. The eight states where the gestation stall ban is currently in effect, Ohio’s ban is effective in 2026, account for less than three percent of the USA pig inventory [31]. However, in California the law includes retail sales of pork produced in other states. California accounts for nearly 15% of domestic pork consumption and 99.8% of the pork consumed in California comes from outside the state [32]. Understanding pig behavior, providing appropriate housing conditions, and ensuring proper animal care is important to everyone involved in modern animal agriculture. Research in this area focuses on assessing animal wellbing indicators, studying behavior patterns, improving housing systems, and developing wellbing assessment protocols and guidelines.

Another significant change accompanying the transition from a commodity industry to a food industry has been reduced reliance on family labor. Today’s capital and technology intensive farms and plants have created a greater demand for skilled, full-time workers [33]. In both the United States and Canada labor challenges are caused by long-term demographic trends of declining and aging rural populations make attracting and retaining domestic workers a challenge [33,34]. In the United States the current visa programs for foreign workers are not well suited for jobs providing year around animal care [33].

Pork producers in the USA and Canada continue to adopt advanced technologies and production practices to increase efficiency [35]. Improving production efficiency through genetic improvement, nutrition and feeding technology has been a primary focus in efforts to reduce cost of production [8]. Genetics and nutrition will continue to advance in the future. The adoption of gene editing tools that can introduce precise genetic modifications show promise in advancing all aspects of pork production [35,36]. As we continue to progress in the mechanistic understanding of nutrigenetics, nutrigenomics and reproduction, advancements in production technology will follow.

Advancements in technology and automation will play a significant role in the future of pork production. Automation of labor intensive practices will be one way to address the shortage of farm workers. Specifically, the pork industry is expected to more readily adopt new technologies around precision farming, sensors, artificial intelligence, and robotics [37]. Monitoring animals and environmental conditions in real-time can help farmers optimize pig production, improve welfare, and make informed decisions.

## CONCLUSION

The pig production industry in the United States and Canada is a vital contributor to the agricultural sectors of both nations. The USA, with its larger population and market size, boasts a prominent position as the third-largest global producer of pork, while Canada follows as the seventh-largest. The symbiotic relationship between the two countries, fostered by trade agreements such as NAFTA and USMCA, has led to tightly integrated production systems and aligned pork prices. Large-scale, modern, and intensive production systems dominate, showcasing a commitment to efficiency, productivity, and collaboration across the supply chain. The availability of feed grains, where the USA and Canada excel as major producers, provides a cost advantage to pig producers. Regulatory frameworks ensure rigorous standards in food safety, animal welfare, and environmental stewardship, bolstering the industry's global competitiveness. The well-established distribution networks and export markets contribute significantly to the industry's success, with both countries exploring opportunities in emerging markets, particularly in Asia. However, challenges such as changing consumer preferences, disease outbreaks, and evolving regulations necessitate continuous adaptation and innovation. Looking ahead, the industry will respond to consumer demands for sustainability, ethical production, and reduced carbon footprints. Advanced technologies, including precision farming, artificial intelligence, and robotics, are anticipated to play a pivotal role in increasing efficiency and ensuring responsible practices. Pig production in the United States and Canada reflects a dynamic and evolving industry, responding to global demands, consumer preferences, and technological advancements. Through a commitment to quality, safety, and sustainability both nations will remain key players in the global pork market, with a promising trajectory for continued growth and innovation.

## Figures and Tables

**Table 1:** Ten states and provinces account for more than seventy percent of the 2021 pig crop of the United States and Canada

Rank	State/Province	Pig crop, head
1	Iowa	23,063,000
2	North Carolina	17,985,000
3	Minnesota	12,816,000
4	Illinois	11,555,900
5	Missouri	9,692,000
6	Nebraska	8,949,000
7	Manitoba	8,719,200
8	Oklahoma	8,345,000
9	Ontario	7,918,200
10	Quebec	7,228,200
	United States	133,359,200
	Canada	30,210,900

USDA [2]; Statistics Canada [3].

**Table 2:** Largest pork producers in the United States and Canada in 2022

Rank	Producer		Sows, head
1	Smithfield Foods	USA	930,000
2	Pipestone System	USA	288,000
3	Triumph Foods	USA	426,640
4	Seaboard Foods	USA	335,000
5	Iowa Select Farms	USA	242,500
6	Carthage System	USA	178,600
7	Prestage Farms	USA	178,000
8	The Maschoffs	USA	176,000
9	AMVC Management Services	USA	150,500
10	Olymel	Canada	134,000
11	Hylife (Charoen Pokphand Foods)	Canada	132,000
12	Clemens Country View Family Farms	USA	105,000

Genesus Inc [9].

**Table 3:** Pork Exports from the USA and Canada in 2022

Country	USA Pork Exports		Canadian Pork Exports	
	
	Value (USD)	Volume (Metric tons)	Value (USD)	Volume (Metric tons)
Mexico	$2,041,829,000	968,272	$289,947,847	192,179
Japan	$1,477,947,000	357,023	$927,657,436	239,814
China	$1,363,276,000	535,570	$572,847,618	278,997
USA	-	-	$1,256,651,807	409,702
Canada	$867,258,00	186,712	-	-
South Korea	$609,546,00	174,957	$151,120,000	60,451
Philippines	$134,866,000	45,456	$220,688,210	125,399

USDA [1]; Canada Pork [10].
